# Cautious Application of Pleural N-Terminal Pro-B-Type Natriuretic Peptide in Diagnosis of Congestive Heart Failure Pleural Effusions among Critically Ill Patients

**DOI:** 10.1371/journal.pone.0115301

**Published:** 2014-12-12

**Authors:** Jiann-Horng Yeh, Chun-Ta Huang, Chia-Hsiung Liu, Sheng-Yuan Ruan, Yi-Ju Tsai, Ying-Chun Chien, Ching-Yao Yang, Chun-Kai Huang, Chia-Lin Hsu, Lu-Cheng Kuo, Pei-Lin Lee, Shih-Chi Ku, Ping-Hung Kuo, Chong-Jen Yu, The HINT Study Group

**Affiliations:** 1 Department of Neurology, Shin Kong Wu Ho-Su Memorial Hospital, Taipei, Taiwan; 2 Department of Internal Medicine, National Taiwan University Hospital, Taipei, Taiwan; 3 Department of Traumatology, National Taiwan University Hospital, Taipei, Taiwan; 4 Graduate Institute of Clinical Medicine, National Taiwan University, Taipei, Taiwan; 5 Department of Surgery, National Taiwan University Hospital, Taipei, Taiwan; 6 School of Medicine, College of Medicine, Fu-Jen Catholic University, New Taipei, Taiwan; 7 Hospitalist in National Taiwan University Hospital, Taipei, Taiwan; University of Buenos Aires, Faculty of Medicine, CardiovascularPathophysiology Institute, Argentina

## Abstract

**Background and Objective:**

Several studies on diagnostic accuracy of pleural N-terminal pro-B-type natriuretic peptide (NT-pro-BNP) for effusions from congestive heart failure (CHF) conclude that pleural NT-pro-BNP is a useful biomarker with high diagnostic accuracy for distinguishing CHF effusions. However, its applicability in critical care settings remains uncertain and requires further investigations.

**Methods:**

NT-proBNP was measured in pleural fluid samples of a prospective cohort of intensive care unit patients with pleural effusions. Receiver operating characteristic curve analysis was performed to determine diagnostic accuracy of pleural NT-proBNP for prediction of CHF effusions.

**Results:**

One hundred forty-seven critically ill patients were evaluated, 38 (26%) with CHF effusions and 109 (74%) with non-CHF effusions of various causes. Pleural NT-proBNP levels were significantly elevated in patients with CHF effusions. Pleural NT-pro-BNP demonstrated the area under the curve of 0.87 for diagnosing effusions due to CHF. With a cutoff of 2200 pg/mL, pleural NT-proBNP displayed high sensitivity (89%) but moderate specificity (73%). Notably, 29 (27%) of 109 patients with non-CHF effusions had pleural NT-proBNP levels >2200 pg/mL and these patients were more likely to experience septic shock (18/29 vs. 10/80, P<0.001) or acute kidney injury (19/29 vs. 9/80, P<0.001).

**Conclusions:**

Among critically ill patients, pleural NT-proBNP measurements remain a useful diagnostic aid in evaluation of pleural effusions. However, patients with non-CHF effusions may exhibit high pleural NT-proBNP concentrations if they suffer from septic shock or acute kidney injury. Accordingly, it is suggested that clinical context should be taken into account when interpreting pleural NT-proBNP values in critical care settings.

## Introduction

Pleural effusions are common manifestations of a variety of diseases and congestive heart failure (CHF) is the leading cause of pleural effusions. Traditionally, the well-established criteria of Light are usually used to determine whether the effusion is a transudate or an exudate in the first step of evaluating patients with pleural effusions [1]. However, since the criteria were developed to detect exudates with high sensitivity for not overlooking underlying causes, such as infections and malignancies, they mislabeled approximately 25% of transudates due to CHF as exudates, particularly after the diuretic therapy [2], [3]. Thus, a strategy to diagnose CHF effusions more accurately would possibly avoid unnecessary invasive diagnostic procedures and save medical expenses.

N-terminal pro-B-type natriuretic peptide (NT-proBNP) and its C-terminal counterpart, BNP, are derived from the precursor peptide, namely proBNP. The precursor proBNP is synthesized mainly in heart ventricles and is stimulated by increased tension or stretching of the ventricular wall. Serum NT-proBNP measurement is a biomarker of cardiac dysfunction and has been proven to be a useful tool in diagnosis of CHF [4]. In 2004, Porcel et al. first studied the potential usefulness of pleural NT-proBNP levels for diagnostic purposes and found that its levels could help discriminate CHF effusions from non-CHF effusions [5]. Subsequently, several lines of evidence has demonstrated that NT-proBNP in the pleural effusion is a very useful biomarker with excellent diagnostic accuracy for distinguishing pleural effusions of CHF [6]. However, none of these studies specifically included critically ill patients. It is well known that elevated serum NT-proBNP concentrations are also observed in patients with severe sepsis, septic shock, and acute respiratory distress syndrome [7], [8]. Thus, the utilization of pleural NT-proBNP in diagnosis of pleural effusions attributable to CHF in this patient population may be questionable.

Accordingly, the present study aimed to investigate the applicability of NT-proBNP in pleural effusions in discriminating between CHF and non-CHF effusions among critically ill patients.

## Methods

### Patients

The present study was carried out prospectively between September 2012 and July 2014 at National Taiwan University Hospital in Taipei, Taiwan. During this time, pleural effusion samples from consecutive patients who had undergone thoracentesis in the medical intensive care unit (ICU) were studied. When repeated thoracentesis were required, only the first episode was included. In addition, patients with effusions of uncertain etiology or effusions with more than one possible cause were excluded from the analysis. The decision to undertake thoracentesis was made by the intensivists in charge of patients.

### Ethics statement

This study was approved by the Research Ethics Committee of National Taiwan University Hospital, and all patients or the next of kin gave written informed consent.

### Laboratory measurements

Biochemical analysis (total protein, lactate dehydrogenase, and glucose), bacterial, fungal and mycobacterial culture, Gram stain, acid-fast bacilli smear, and cytological examinations were performed for all pleural effusion samples shortly after thoracentesis. Meanwhile, concentrations of albumin, total protein and lactate dehydrogenase in the serum were measured by standard methods, and the upper limit of normal for serum lactate dehydrogenase was 271 IU/L at our hospital. In addition, a 5-mL sample of pleural effusion was collected in the ethylenediaminetetraacetic acid tube, which was centrifuged at 4°C. Subsequently, the supernatant was frozen at −70°C until the NT-proBNP assay was performed. The storage stability of pleural NT-proBNP has been demonstrated in this study (S1 Figure).

Pleural NT-proBNP levels were determined in duplicate using the commercially available enzyme immunoassay kit (SK-1204, Biomedica Slovakia, s.r.o., Bratislava, Slovakia) following the manufacturer's instructions. The test has intra- and inter-assay coefficients of variation of 4–7% and 9–12%, respectively, and a measuring range of 0–640 fmol/mL. Using the manufacturer-provided conversion factor, the upper limit of this range was converted to 5400 pg/mL (1 fmol/mL  = 8.475 pg/mL). Samples with values above the upper limit of detection were assayed again after dilution. The investigators involved in NT-proBNP measurements were unaware of clinical data.

### Data collection

Patient records were evaluated in detail to obtain demographics, comorbidities, and events during the ICU stay. The presence of uni- or bilateral pleural effusions was confirmed by chest ultrasound examination. The causes of effusions were determined based on predefined criteria by two independent investigators blinded to pleural NT-proBNP levels. If there was discordance, it was resolved by consensus. Specifically, diagnosis of CHF was made according to clinical symptoms, medical history, physical examination, chest radiography and response to diuretic therapy, and confirmed by echocardiographical evidence of left ventricular systolic dysfunction, severe valvular disease or severe left ventricular diastolic dysfunction. The cause of pleural effusions was ascribed to hypoalbuminemia if the serum albumin level was ≦1.8 g/dL and there were bilateral effusions with no pulmonary parenchymal disease. Malignant pleural effusions were diagnosed if malignant cells were detected on cytological examination of pleural effusions or biopsy specimens. Parapneumonic effusions were associated with the clinical and radiological diagnosis of bacterial pneumonia. Other causes of pleural effusions were defined by clear clinical pictures or established diagnostic criteria. In the present study, the terms transudate or exudate were used to label effusions based on their etiologies rather than on criteria of Light et al. [1].

### Statistical analysis

Quantitative variables were presented as means and standard deviations, and were analyzed by the independent sample t-test. Qualitative variables were presented as numbers and percentages, and were analyzed by the Fisher exact or χ^2^ tests. To determine the discriminative power of various cutoffs of NT-proBNP, the receiver operating characteristic (ROC) curve was drawn and the area under the curve (AUC) was calculated. The best cutoff was determined by the Youden index, and the sensitivity, specificity, positive likelihood ratio, and negative likelihood ratio were calculated according to standard formulae. A two-tailed P value of <0.05 was considered statistically significant. All data were analyzed using SPSS for Windows version 15.0 (SPSS Inc., Chicago, IL).

## Results

### Patient characteristics

**Figure 1:**
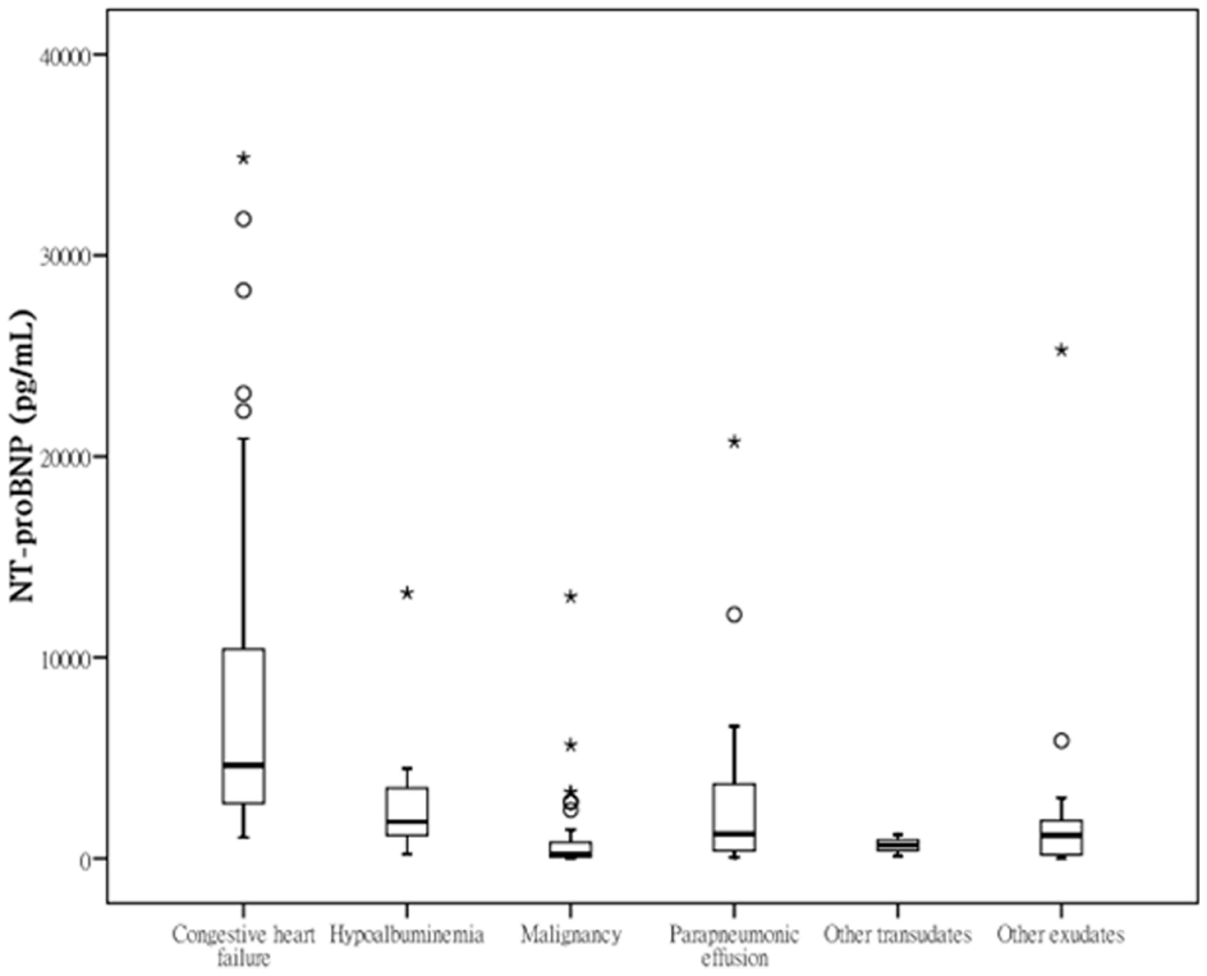
Boxplots showing the minimum, 25th, 50th, and 75th percentiles, and the maximum pleural N-terminal pro-B-type natriuretic peptide (NT-proBNP) levels, by etiologies of pleural effusions. Outliers (open circles) and extremes (stars) are plotted separately.

During the study period, pleural effusion samples were collected from 147 medical ICU patients with a mean age of 71 years. Depending on the causal disease of the effusions, 67 were labeled as transudates and 80 were labeled as exudates (Table 1). The clinical and biochemical features of the study population according to the etiology of pleural effusions are detailed in Table 2. Patients with CHF effusions more often had history of CHF and chronic kidney disease, and had significantly lower pleural effusion levels of total protein and lactate dehydrogenase. The mean NT-proBNP concentration of CHF effusions was significantly higher than that of non-CHF effusions (8863 pg/mL vs. 2062 pg/mL, P<0.001). Fig. 1 displays the median values of NT-proBNP based on the specific etiologies.

**Table 1:** Etiologies of pleural effusions.

Categories of effusions	Patient No. (n = 147)
Transudates	67
Congestive heart failure	38
Hypoalbuminemia	18
Others*	11
Exudates	80
Malignancy	34
Parapneumonic effusion	27
Others†	19

*Effusions due to atelectasis, hepatic hydrothorax, or nephrotic syndrome.

† Effusions due to chylothorax, connective tissue disease, hypothyroidism, lymphangioleiomyomatosis, subphrenic abscess, tuberculosis, or uremia.

**Table 2:** Characteristics of the study population.

Characteristics	Congestive heart failure (n = 38)	Non-congestive heart failure (n = 109)	P value
Age, years	75±12	70±14	0.089
Male sex	24 (63)	66 (61)	0.776
APACHE II score*	22±8	23±8	0.430
Intensive care unit mortality	10 (26)	26 (24)	0.761
Past history			
Congestive heart failure	28 (74)	11 (10)	<0.001
Chronic kidney disease	21 (55)	18 (17)	<0.001
Diabetes mellitus	17 (45)	42 (39)	0.502
Intensive care unit events			
Septic shock	6 (16)	28 (26)	0.213
Acute kidney injury	7 (18)	28 (26)	0.365
Bilateral effusions	26 (68)	40 (37)	0.001
Pleural effusion			
Exudates by Light's criteria	6 (16)	84 (77)	<0.001
Glucose, mg/dL	151±29	134±45	0.033
Total protein, g/dL	1.8±0.8	3.1±1.3	<0.001
Lactate dehydrogenase, U/L	118±118	450±852	<0.001
NT-proBNP, pg/mL†	8863±9058	2062±3769	<0.001

Data are presented as No. (%) or mean ± standard deviation.

*APACHE, Acute Physiology and Chronic Health Evaluation.

† NT-proBNP, N-terminal pro-B-type natriuretic peptide.

### Operating characteristics of pleural NT-proBNP levels

**Figure 2:**
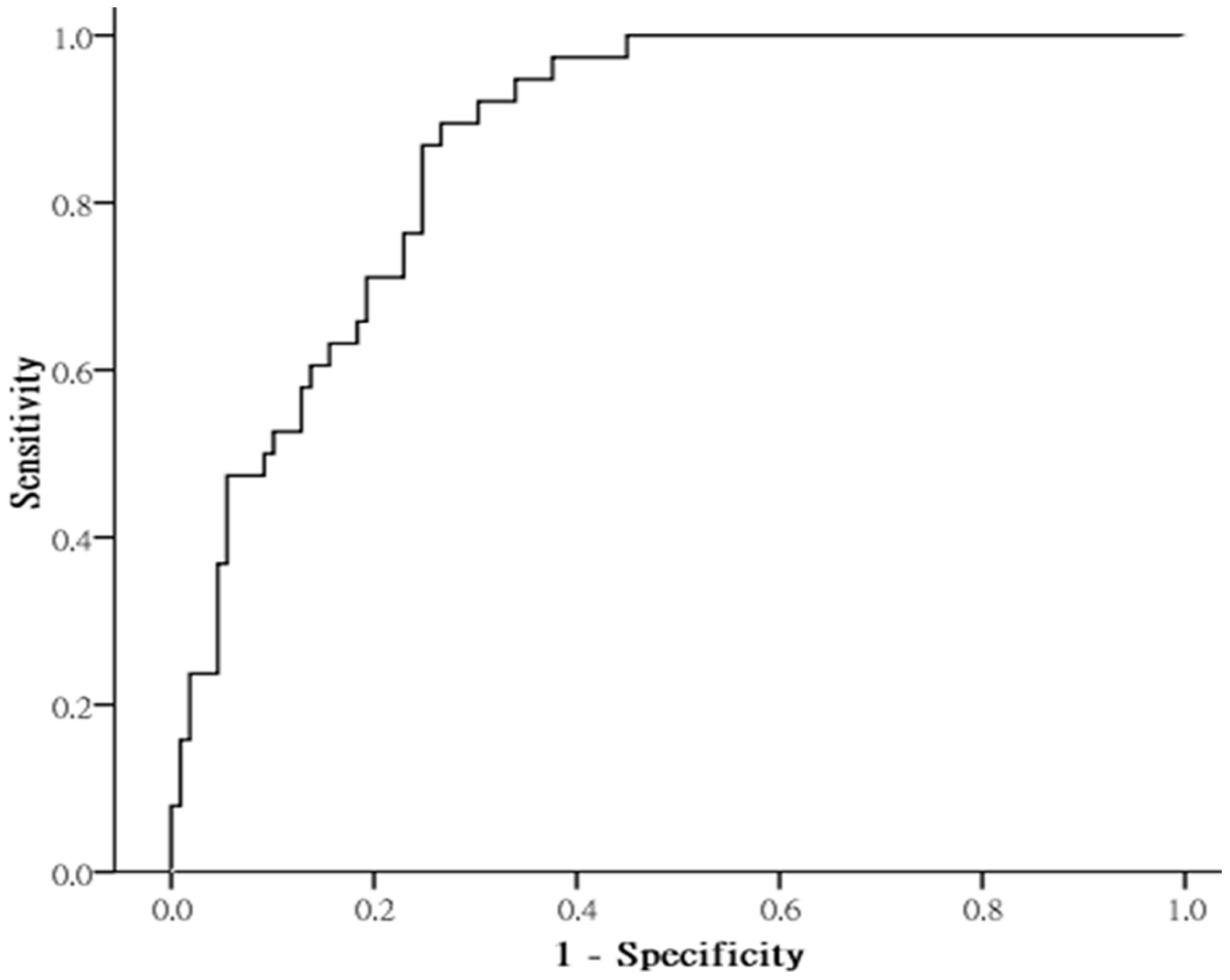
Receiver operating characteristic curve of pleural N-terminal pro-B-type natriuretic peptide levels comparing patients with pleural effusions caused by congestive heart failure to those with pleural effusions attributable to other reasons. The area under the curve was 0.87 (95% confidence interval 0.81–0.92).

In distinguishing between patients with CHF effusions (n = 38) and patients with non-CHF effusions (n = 109), the AUC for pleural NT-proBNP was 0.87 (Fig. 2). The ROC curve analysis selected 2200 pg/mL as the best cutoff value in the pleural effusion (sensitivity 89%, specificity 73%) to establish such discrimination. Table 3 displays the sensitivity, specificity, and positive and negative likelihood ratios of pleural NT-proBNP for the diagnosis of CHF effusions.

**Table 3:** Diagnostic information for pleural N-terminal pro-B-type natriuretic peptide concentrations in the diagnosis of pleural effusions caused by congestive heart failure.

Pleural NT-proBNP cutoffs, pg/mL*	Sensitivity %	Specificity %	Positive likelihood ratio	Negative likelihood ratio
>1000	100	55	2.2	0.00
>1500	92	66	2.8	0.08
>2200†	89	73	3.4	0.14
>4000	58	87	4.5	0.48

*NT-proBNP, N-terminal pro-B-type natriuretic peptide.

† Optimal cutoff value determined by Youden index.

### Classification of mislabeled CHF effusions

In all, 6 (16%) of the 38 patients with CHF effusions would have been misclassified by the Light's criteria (Table 2). Five of these effusions had pleural NT-proBNP levels greater than the best cutoff of 2200 pg/mL. On the other hand, pleural NT-proBNP levels of >2200 pg/mL were observed in 29 (27%) of the 109 non-CHF effusions. These patients were more likely to have septic shock (18/29 vs. 10/80, P<0.001) or acute kidney injury (19/29 vs. 9/80, P<0.001) than those having non-CHF effusions with NT-proBNP levels ≦2200 pg/mL. There were no differences in age, gender distribution, or history of CHF and chronic kidney disease between patients with non-CHF effusions having pleural NT-proBNP levels of >2200 and ≦2200 pg/mL.

## Discussion

For the first time, this study evaluated the effectiveness of pleural NT-proBNP in diagnosis of CHF pleural effusions among ICU patients. We demonstrated that NT-proBNP levels in pleural effusions were significantly higher in patients with CHF effusions as compared to the others. For diagnosing CHF effusions, using the ROC curve analysis, pleural NT-proBNP showed an AUC of 0.87, indicating its good, although not excellent, diagnostic accuracy, and an NT-proBNP level of >2200 pg/mL displayed a sensitivity of 89% and a specificity of 73%. Of note, in patients with non-CHF effusions, more than one fourth (27%) of them had pleural NT-proBNP levels of >2200 pg/mL and these patients were more likely to experience septic shock and acute kidney injury than the others.

In the past decade, a number of studies assessing the diagnostic utility of pleural NT-proBNP in CHF effusions have been reported [5], [9]–[17]. A recent meta-analysis summarizing these studies showed that the pooled sensitivity and specificity were both 94% [18]. In this study, an NT-proBNP level of >2200 pg/mL had a similar high sensitivity but a significantly lower specificity for predicting CHF effusions in the critically ill population. Spectrum effects and population bias may, at least in part, explain the discrepancy in results. Spectrum effects refer to the phenomenon that the performance of a diagnostic test varies in different clinical settings. Testing is not done in the clinically relevant population, but rather a limited subset [19], [20]. Some previous studies specifically selected patients with pleural effusions due to CHF and patients with non-CHF effusions as their study population [9], [13]–[15], [17]. The existence of spectrum effects can result in overestimation of the sensitivity and specificity of the diagnostic test [19]. Population bias refers to the generalizability of a diagnostic test to a wider population [21]. The majority of prior studies did not report any comorbid conditions of the study patients [5], [9], [11], [13]–[17]; thus, this may hamper the generalization of the results to the population of our interest. Taken together, the present study adds to the current knowledge base and indicates that it remains reliable to exclude diagnosis of CHF effusions in the ICU setting when the pleural NT-proBNP value is below the cutoff point. However, on the contrary, it should be cautious to ascribe pleural effusions to CHF if an NT-proBNP level in pleural effusions is above the cutoff.

In terms of ROC curve analysis, previous studies have demonstrated excellent diagnostic accuracy of pleural NT-proBNP in diagnosis of CHF effusions. Even more surprisingly, Tomcsanyi, Kolditz, and Liao et al. reported an AUC of 0.98–1 [9], [10], [13]. In our study, pleural NT-proBNP was also good at differentiating CHF effusions from non-CHF effusions, albeit at a lower AUC of 0.87. In addition to spectrum effects and population bias, several patient factors that may influence NT-proBNP levels, such as age, gender, renal function, thyroid function, anemia, and body habitus, should be taken into account [22], [23]. No studies to date have specifically tackled this issue. Accordingly, it is suggested that future studies have to clarify the confounding effects of these clinical factors.

A major difference between previous studies and ours is that we specifically enrolled critically ill patients and found a moderate specificity of pleural NT-proBNP in diagnosing CHF effusions. Twenty-seven percent of patients with non-CHF effusions had pleural NT-proBNP levels of >2200 pg/mL. A significantly higher proportion of these patients suffered from septic shock and acute kidney injury as compared to those having pleural NT-proBNP levels of ≦2200 pg/mL. The role of elevated NT-proBNP levels in cardiovascular diseases is well established [24]. However, comparable increase of NT-proBNP was observed in patients septic shock and CHF [7], [25]. Pathophysiological mechanisms other than myocardial dysfunction may contribute to increased NT-proBNP levels in septic patients. NT-proBNP is mainly cleared by the kidneys and it is well known that concentrations of NT-proBNP are increased in acute kidney injury and chronic kidney disease [26], [27]. Moreover, a few studies have demonstrated a strong correlation between pleural and serum NT-proBNP values [9]–[12]. Taken together, it is anticipated that even among patients with non-CHF effusions, they will have higher NT-proBNP levels in pleural effusions if they get septic shock or acute kidney injury. Thus, the finding herein is pathophysiologically sound and raises a caveat concerning the interpretation of pleural NT-proBNP in critically ill patients.

Light's criteria remain the reference standard for distinguishing transudates from exudates and are always applied in the first step in the evaluation of pleural effusions [1]. The criteria of Light exhibit high ability to exclude the possibility of exudates; however, pleural effusions in CHF may meet the Light's exudative criteria in approximately one fourth of the cases [2], [3]. In the present study, 6 of the 38 patients with CHF effusions were labeled as exudates, but almost all 38 patients had pleural NT-proBNP levels above the best cutoff. Given the high sensitivity and moderate specificity of pleural NT-proBNP in diagnosis of CHF effusions among our ICU population, this study suggests a somewhat dissimilar strategy to the one proposed by other investigations [5], [9]–[15], [17]. In the critically ill setting, Light's criteria are most useful in excluding exudative pleural effusions and pleural NT-proBNP measurements are powerful in ruling out CHF effusions. Accordingly, the two diagnostic tools are complementary to each other in assessment and management of undiagnosed pleural effusions among ICU patients.

One limitation of the present study needs to be addressed. The kit for assaying NT-proBNP in this study differs from the commonly used electrochemiluminescence immunoassay for NT-proBNP offered by Roche Diagnostics; however, the diagnostic cutoff of NT-proBNP we established is well within the range reported in other studies using the Roche assay [5], [10], [12], [14], [16], [17].

In conclusion, the diagnostic accuracy of pleural NT-proBNP in differentiating between CHF and non-CHF effusions remains good in critically ill patients, although its diagnostic specificity is moderate. Pleural NT-proBNP levels of ≦2200 pg/mL virtually exclude diagnosis of CHF effusions; however, NT-proBNP levels of >2200 pg/mL in pleural effusions should be interpreted with caution. Patients with non-CHF effusions may have pleural NT-proBNP levels above the best cutoff if they experience septic shock or acute kidney injury. This study suggests that pleural NT-proBNP and Light's criteria should be complementary to each other in the diagnostic approach of pleural effusions and clinical context should be taken into account when interpreting pleural NT-proBNP values in the ICU setting.

## Supporting Information

S1 Figure **Scatterplot with linear regression showing the storage stability of pleural N-terminal pro-B-type natriuretic peptide (NT-proBNP).** Thirty pleural effusion samples were assayed for NT-proBNP both immediately after sample collection and at approximately one month after storage at −70°C.
(TIF)
